# The efficacy of different seed priming agents for promoting sorghum germination under salt stress

**DOI:** 10.1371/journal.pone.0245505

**Published:** 2021-01-19

**Authors:** Xiaofei Chen, Ruidong Zhang, Yifan Xing, Bing Jiang, Bang Li, Xiaoxue Xu, Yufei Zhou

**Affiliations:** 1 College of Agronomy, Shenyang Agricultural University, Shenyang, Liaoning, China; 2 Institute of Economic Crop, Shanxi Academy of Agricultural Sciences, Fenyang, Shanxi, China; 3 Tongliao Agricultural Technology Extension Station, Tongliao, Inner Mongolia, China; Department of Agronomy, University of Agriculture, Faisalabad, PAKISTAN

## Abstract

Sorghum [*Sorghum bicolor* (L.) Moench] seed germination is sensitive to salinity, and seed priming is an effective method for alleviating the negative effects of salt stress on seed germination. However, few studies have compared the effects of different priming agents on sorghum germination under salt stress. In this study, we quantified the effects of priming with distilled water (HP), sodium chloride (NaCl), potassium chloride (KCl), calcium chloride (CaCl_2_), and polyethylene glycol (PEG) on sorghum seed germination under 150 mM NaCl stress. The germination potential, germination rate, germination index, vigor index, root length, shoot length, root fresh weight, shoot fresh weight, root dry weight, and shoot dry weight were significantly reduced by salt stress. Different priming treatments alleviated the germination inhibition caused by salt stress to varying degrees, and 50 mM CaCl_2_ was the most effective treatment. In addition, the mitigation effect of priming was stronger on root traits than on shoot traits. Mitigation efficacy was closely related to both the type of agent and the concentration of the solution. Principal component analysis showed that all concentrations of CaCl_2_ had higher scores and were clearly distinguished from other treatments based on their positive effects on all germination traits. The effects of the other agents varied with concentration. The priming treatments were divided into three categories based on their priming efficacy, and the 50, 100, and 150 mM CaCl_2_ treatments were placed in the first category. The 150 mM KCl, 10% PEG, HP, 150 mM NaCl, 30% PEG, and 50 mM KCl treatments were placed in the second category, and the 100 mM NaCl, 100 mM KCl, 20% PEG, and 50 mM NaCl treatments were least effective and were placed in the third category. Choosing appropriate priming agents and methods for future research and applications can ensure that crop seeds germinate healthily under saline conditions.

## Introduction

Seed germination is a critical stage of the plant life cycle. During germination, the dormant seed becomes highly active and eventually becomes a healthy seedling [1,2]. Seed germination begins with water absorption and is accompanied by degradation of macromolecular substances, repair of genetic materials, and expansion of the embryo and endosperm, eventually leading to rupture of the seed coat and endosperm and the production of a prominent radicle [3,4]. Healthy seed germination strongly influences early seedling establishment and final yield [5]. However, germination is susceptible to environmental stresses such as salt stress [6,7]. Over 900 million hectares, approximately 20% of the world’s total agricultural land, are affected by salt, and it has become an increasingly serious problem in agricultural production [8].

Although all stages of plant growth are affected by salt stress, the seed germination stage is the most sensitive [9]. Seed germination is inhibited by high concentrations of sodium and chloride ions, mainly because they reduce the osmotic potential of the surrounding environment, thereby suppressing seed imbibition and embryo growth [1,10,11]. In addition, ion toxicity also destroys macromolecular substances and affects energy utilization and metabolism during germination [12]. Numerous studies have shown that salt stress can significantly reduce seed vigor and inhibit germination and early seedling growth in many species [13–15]. Effective methods for promoting seed germination in saline conditions are therefore needed, and they are especially crucial for crop production on saline-alkali land [12].

At present, seed coating and seed priming are the main two methods for enhancement of sorghum seed germination under salt stress conditions. Seed priming is one of the most frequently used techniques, and is the method mainly employed by farmers [16]. This pre-sowing treatment allows partial hydration of seeds without causing full radicle protrusion. Seed priming usually involves the first two stages of seed germination (imbibition and activation), and it eventually leads to a higher seed germination rate and improves the uniformity of germination [17]. Seed priming technology is used to improve germination under both favorable and unfavorable conditions [18], and its effects may be greater under adverse conditions than under favorable conditions [19–21]. Hydropriming and osmopriming are the two most common priming methods [22]. During hydropriming, seeds are soaked in water, which promotes seed germination to some extent [23]. Many studies have reported that hydropriming is effective in promoting seed germination under salt stress [24–26]. Polyethylene glycol (PEG) is a large molecular weight penetrant with wide commercial application [22], and studies have reported that seed priming with PEG promotes germination under salt stress [27,28]. In addition, ions such as potassium, sodium, and calcium are used for osmopriming through treatment with NaCl, KCl, CaCl_2_, etc. These compounds are relatively inexpensive and easy to obtain [22], and their ability to improve seed germination under salt stress has been reported in several studies [29–31]. Although there are many reports of the beneficial effects of these seed priming methods during salt stress, few studies have investigated whether the above-mentioned agents improve sorghum seed germination under salt stress. Some other priming agents have also been used for this purpose, such as nano-iron oxide [32], salicylic acid, kinetin and gibberellic acid [33]. Furthermore, studies comparing the relative effects of different priming agents on sorghum germination under salt stress have rarely been reported.

Sorghum [*Sorghum bicolor* (L.) Moench] is one of the most important cereal crops in the world following maize, rice, wheat and barley. Although sorghum is highly stress resistant, it is sensitive to salt during germination, and salt exposure can limit early seedling establishment and reduce final yields [34]. The purposes of this study are (1) to clarify the role of different priming agents on sorghum seed germination under salt stress and (2) to determine which priming agent and concentration has the best effect on sorghum germination under salt stress. Our results provide insight into the mitigation of salt stress effects on sorghum germination.

## Materials and methods

### Plant material

Sorghum seeds [*Sorghum bicolor* (L.) Moench] of the Liaoza15 variety were harvested in 2017 and provided by the Liaoning Academy of Agricultural Sciences for use in this study. Preliminary experiments demonstrated that it showed moderate germination sensitivity to 150 mM salt stress. The germination rate of these seeds is above 90% under favorable conditions. All seeds were stored in a 4°C refrigerator for later use.

### Experimental design

The study was conducted in the sorghum physiology laboratory of Shenyang Agricultural University in 2018. Uniform sorghum seeds were selected and surface sterilized with a 5% NaClO solution for 10 min. The seeds were then washed five times with distilled water and surface dried [35]. First, two treatments (NPN, no priming and no stress; NPS, no priming and salt stress) were used to determine the effect of salt stress on sorghum seed germination. Next, sterilized seeds were primed with HP, sodium chloride solutions (NaCl at 50, 100, and 150 mM), potassium chloride solutions (KCl at 50, 100, and 150 mM), calcium chloride solutions (CaCl_2_ at 50, 100, and 150 mM), and polyethylene glycol 6000 solutions (PEG 6000 at 10, 20, 30% w/v) for 12 h at 25°C in the dark with continuous gentle stirring. The ratio of seeds to solution was 1:5 (w/v). After priming, the seeds were air dried to their original moisture content (13%, w/w), and unprimed seeds were used as controls (NPS) [36]. For each treatment, seeds were placed into petri dishes with double-layer sterile filter paper, 10 ml of treatment solution (150 mM NaCl) was added, and the seeds were cultivated in a dark growth chamber at 25 ± 1°C and approximately 70% relative humidity for 10 d (days). To maintain a constant treatment solution concentration, the 150 mM NaCl solution in the petri dishes of each treatment was refreshed daily. Each treatment was replicated three times with 50 seeds per dish, and the experiment was arranged in a completely randomized design.

The numbers of germinated seeds were recorded every day. After 10 d of germination, five seedlings were randomly selected from each replicate for measurement of shoot length (SL), shoot fresh weight (SFW), shoot dry weight (SDW), root length (RL), root fresh weight (RFW), and root dry weight (RDW), and the mean was calculated. SL and RL were measured with a ruler. Shoot and root tissues were dried to a constant weight at 80°C in an oven. Fresh and dry weights were measured using analytical balances (Mettler Toledo, Switzerland). The germination rate (GR), germination potential (GP), germination index (GI), and vigor index (VI) were calculated according to the following formulae.

$$\text{GR}=\frac{\text{Total}\;\text{number}\;\text{of}\;\text{germinated}\;\text{seeds}\;\text{on}\;\text{day}\;10}{\text{Total}\;\text{number}\;\text{of}\;\text{seeds}}\times 100\%$$ (1)

$$\text{GP}=\frac{\text{Total}\;\text{number}\;\text{of}\;\text{germinated}\;\text{seeds}\;\text{on}\;\text{day}\;4}{\text{Total}\;\text{number}\;\text{of}\;\text{seeds}}\times 100\%$$ (2)

$$\begin{array}{l} \text{GI}=(\text{number}\;\text{of}\;\text{seeds}\;\;\text{germinated}\;\text{on}\;\text{day}\;1/1\text{)}\\ +\text{(number}\;\text{of}\;\text{seeds}\;\;\text{germinated}\;\text{on}\;\text{day}\;2/2\text{)}\\ +\ldots \\ +\text{(number}\;\text{of}\;\text{seeds}\;\;\text{germinated}\;\text{on}\;\text{day}\;10/10) \end{array}$$ (3)

VI=GI×RFW (4)

[37]

Relative values of the above indicators were calculated as the ratio of the treatment value to the control value.

### Statistical analysis

Origin 2018 (*OriginLab*, Massachusetts, USA) was used to generate a heat map of correlation coefficients among germination traits. One-way analysis of variance (ANOVA) and cluster analysis were performed using SPSS 18.0 software (*SPSS Inc*., Chicago, USA). Cluster analysis classifies all treatments according to their efficacy in promoting germination. Both software packages were used together to calculate principal component analysis scores for each treatment. Duncan’s multiple range test was used to assess significant differences among treatments using SPSS 18.0. Different letters indicate significant differences at p < 0.05.

## Results

### Seed germination traits under salt stress

Seed germination was clearly affected by salt stress in the absence of priming treatment (Table 1). The values of seed germination traits were significantly lower in the salt-stressed NPS treatment than in the unstressed NPN treatment. GP, GR, GI, VI, SL, RL, SFW, RFW, SDW, and RDW were decreased by 38.47%, 19.12%, 43.64%, 86.44%, 56.21%, 63.02%, 45.02%, 75.97%, 38.35%, and 65.32% in NPS compared with NPN, respectively.

**Table 1:** Effects of salt stress on seed germination traits in sorghum.

Treatments	GP (%)	GR (%)	GI	VI	SL (cm)	RL (cm)	SFW (mg)	RFW (mg)	SDW (mg)	RDW (mg)
NPN	86.67a	90.67a	41.22a	16.30a	9.91a	11.44a	82.58a	39.54a	8.97a	2.97a
NPS	53.33b	73.33b	23.23b	2.21b	4.34b	4.23b	45.40b	9.50b	5.53b	1.03b

Notes: NPN: no priming and no stress; NPS: no priming and salt stress; GR: germination rate; GI: germination index; VI: vigor index; SL: shoot length; RL: root length; SFW: shoot fresh weight; RFW: root fresh weight; SDW: shoot dry weight; RDW: root dry weight. Different letters within a column indicate a significant difference at p < 0.05.

### Relative values of seed germination traits under saline conditions

The values of seed germination traits under salt stress in different priming treatments relative to untreated controls are shown in Table 2. All germination traits were higher in the HP treatment than in the unprimed controls, with the exception of SDW. Most germination traits, including RVI and RRL, were higher in the NaCl treatments. No germination traits were lower in the 50KCl and 150KCl treatments, and only GR, SL, SFW, and SDW were lower in the 100KCl treatment. Seed priming with CaCl_2_ increased the values of all germination traits, and the relative values of most traits (RGP, RGI, etc.) were higher in the CaCl_2_ treatments than in the other treatments. Only a few germination traits (RGR, RSFW, and RSDW) were lower in the PEG treatment group.

**Table 2:** Relative values of seed germination traits under saline conditions in different priming treatment groups.

Treatments	RGP	RGR	RGI	RVI	RSL	RRL	RSFW	RRFW	RSDW	RRDW
HP	1.28abc	1.18ab	1.35abc	2.59d	1.29bcde	1.55bc	1.05cde	2.23bcdef	0.90b	1.49d
50NaCl	0.81d	0.67f	0.70e	1.88d	1.26cdef	1.43bc	1.18abcd	2.49abcde	1.01b	1.80bcd
100NaCl	1.17bc	1.09abcd	1.12cd	1.70d	0.96ef	1.39bc	0.82ef	1.65fg	1.00b	1.87bcd
150NaCl	1.09c	1.09abcd	1.17bcd	2.78cd	1.10def	1.50bc	0.96def	2.13cdefg	0.97b	1.83bcd
50KCl	1.06c	1.00bcde	1.13cd	2.47d	1.36bcd	1.46bc	1.07bcde	1.78defg	1.07b	1.87bcd
100KCl	1.06c	0.93cde	1.01d	2.20d	0.94f	1.38bc	0.80ef	1.75efg	0.96b	1.84bcd
150KCl	1.26bc	1.11abcd	1.19bcd	3.35bcd	1.23cdef	1.54bc	1.16abcd	2.33abcdef	1.16b	1.82bcd
50CaCl_2_	1.51a	1.25a	1.59a	5.37a	1.55abc	2.20a	1.30abc	2.96ab	1.47a	2.30b
100CaCl_2_	1.38ab	1.14abc	1.43ab	4.33abc	1.70a	2.26a	1.38ab	2.99a	1.46a	3.13a
150CaCl_2_	1.38ab	1.14abc	1.59a	4.79ab	1.59ab	1.99ab	1.39a	2.59abc	1.52a	2.14bcd
10%PEG	1.06c	0.84ef	1.01d	3.15bcd	1.23cdef	1.74abc	1.14abcd	2.52abcd	1.16b	2.26bc
20%PEG	1.13c	1.00bcde	1.08cd	2.00d	1.07def	1.35c	0.73f	1.51g	0.85b	1.52cd
30%PEG	1.30abc	1.16ab	1.24bcd	2.39d	1.22cdef	1.38bc	0.94def	1.79defg	1.00b	1.72bcd

Notes: RGP: relative germination potential; RGR: relative germination rate; RGI: relative germination index; RVI: relative vigor index; RSL: relative shoot length; RRL: relative root length; RSFW: relative shoot fresh weight; RRFW: relative root fresh weight; RSDW: relative shoot dry weight; RRDW: relative root dry weight. A relative value greater than 1 or less than 1 indicates that the trait is higher or lower than in the control. Different letters within a column indicate significant differences at p < 0.05.

### Correlation among germination traits

**Fig 1:**
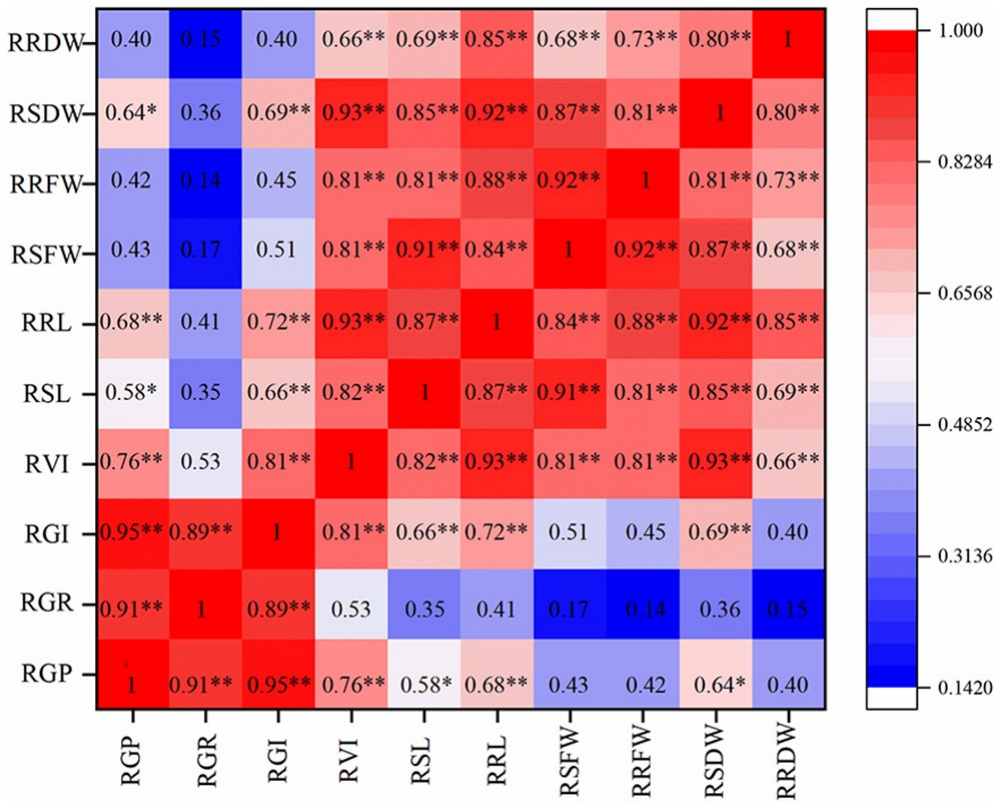
Heat map of correlations among germination traits under saline conditions. RGP: relative germination potential; RGR: relative germination rate; RGI: relative germination index; RVI: relative vigor index; RSL: relative shoot length; RRL: relative root length; RSFW: relative shoot fresh weight; RRFW: relative root fresh weight; RSDW: relative shoot dry weight; RRDW: relative root dry weight. A darker red color indicates a larger correlation coefficient, and a darker blue color indicates a smaller correlation coefficient. * and ** indicate significant correlations at p < 0.05 and p < 0.01, respectively.

There were two groups of highly correlated traits, one comprising RGP, RGR, and RGI and the other comprising RVI, RSL, RRL, RSFW, RRFW, RSDW, and RRDW (Fig 1). The correlations among most germination traits were significant at the p < 0.01 level. RGP was significantly correlated with RVI, RSL, RRL and RSDW, with correlation coefficients of 0.76, 0.58, 0.68, and 0.64, respectively. RGI was highly correlated with RVI, RSL, RRL, and RSDW, with correlation coefficients of 0.81, 0.66, 0.72 and 0.69, respectively. RGR was not significantly correlated with any seedling growth traits (RSL, RRL, etc.), indicating that healthier seedling growth status induced by seed priming was the result of faster germination (i.e., GP and GI), rather than germination rate.

### Principal component analysis

**Fig 2:**
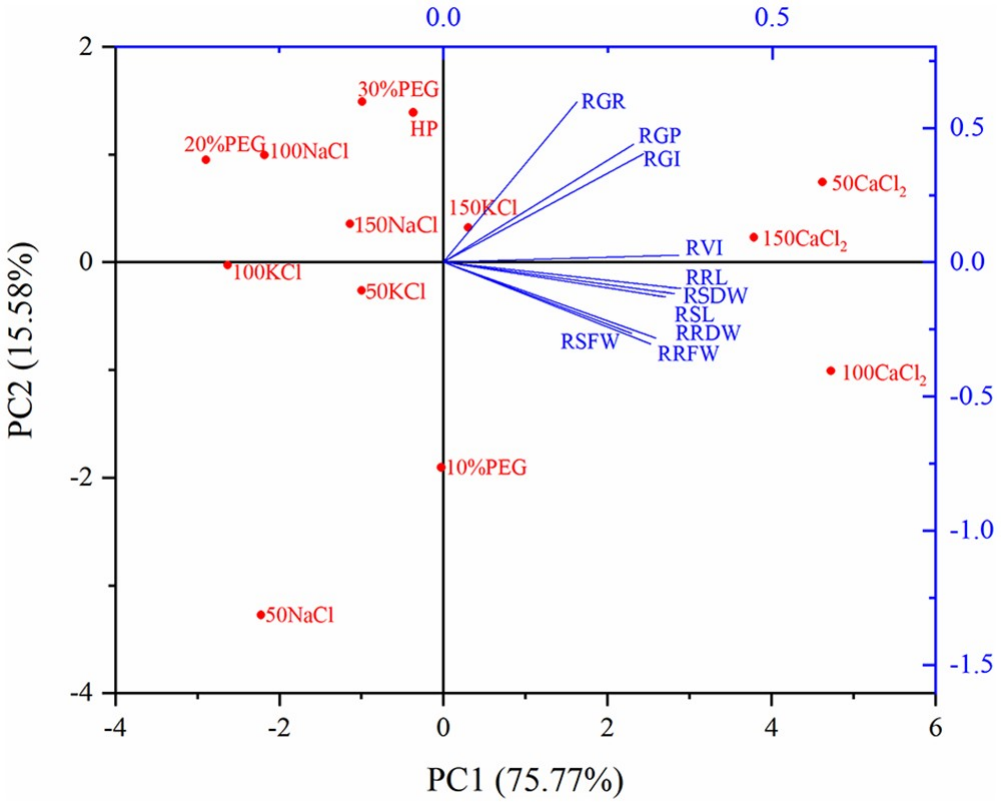
Principle component analysis of germination traits for different seed priming treatments. RGP: relative germination potential; RGR: relative germination rate; RGI: relative germination index; RVI: relative vigor index; RSL: relative shoot length; RRL: relative root length; RSFW: relative shoot fresh weight; RRFW: relative root fresh weight; RSDW: relative shoot dry weight; RRDW: relative root dry weight.

The effects of the different seed priming treatments on sorghum germination traits under salt stress were evaluated by principal component analysis (PCA). PC1 and PC2 explained 75.77% and 15.58% of the overall variation, respectively (Fig 2). The significant effects of the seed priming treatments on germination traits were clearly distributed along the PC1 axis in the order of 100CaCl_2_ > 50CaCl_2_ > 150CaCl_2_ > 150KCl > 10%PEG > HP > 50KCl > 30%PEG > 150NaCl > 100NaCl > 50NaCl > 100KCl > 20%PEG. The CaCl_2_ treatments had markedly higher PC1 scores and were clustered on the right of the PC1 axis; the other treatments with lower scores were observed primarily on the left. PCA also showed higher germination traits in CaCl_2_ treatments compared to the other treatments.

The loading matrix of a given principal component reflected the degree of correlation between the germination traits and the principal component. PC1 had large loadings on all germination traits and showed strong positive correlations. PC2 had large loadings on RGP, RGR and RGI with positive correlations but had negative correlations with seedling growth traits (Table 3). This indicated that PC1 reflected the overall germination status, whereas PC2 reflected the opposite correlations between RGP, RGR, RGI and seedling growth traits.

**Table 3:** Loading matrix for principal components.

	RGP	RGR	RGI	RVI	RSL	RRL	RSFW	RRFW	RSDW	RRDW
PC1	0.834	0.624	0.869	0.969	0.920	0.973	0.882	0.866	0.952	0.755
PC2	0.527	0.758	0.468	0.034	−0.175	−0.123	−0.357	−0.376	−0.143	−0.388

Notes: RGP: relative germination potential; RGR: relative germination rate; RGI: relative germination index; RVI: relative vigor index; RSL: relative shoot length; RRL: relative root length; RSFW: relative shoot fresh weight; RRFW: relative root fresh weight; RSDW: relative shoot dry weight; RRDW: relative root dry weight.

Similar to the results of Table 3, the coefficient matrix of component scores reflects the contribution of germination traits to the principal component score (Table 4). All germination traits had large score coefficients and showed strong positive coefficients, indicating that the higher the score on PC1, the better the effect of the priming treatment on overall germination status of sorghum under salt stress. PC2 had large positive coefficients for RGP, RGR and RGI but negative coefficients for seedling growth traits, indicating that seedling growth traits were not positively correlated with RGP, RGR and RGI, as can also be seen in Table 2. Based on Table 4, the factor score function formulas for PC1 (Y1) and PC2 (Y2) were as follows:

$$\begin{array}{l} \text{Y}1=0.11\text{RGP}+0.082\text{RGR}+0.115\text{RGI}+0.128\text{RVI}+\\ 0.121\text{RSL}+0.128\text{RRL}+0.116\text{RSFW}+0.114\text{RRFW}+\\ 0.126\text{RSDW}+0.1\text{RRDW} \end{array}$$ (5)

$$\begin{array}{l} \text{Y}2=0.338\text{RGP}+0.486\text{RGR}+0.3\text{RGI}+0.022\text{RVI}-\\ 0.112\text{RSL}-0.079\text{RRL}-0.229\text{RSFW}-0.242\text{RRFW}-\\ 0.092\text{RSDW}-0.249\text{XRRDW} \end{array}$$ (6)

**Table 4:** Coefficient matrix of component scores.

	RGP	RGR	RGI	RVI	RSL	RRL	RSFW	RRFW	RSDW	RRDW
PC1	0.110	0.082	0.115	0.128	0.121	0.128	0.116	0.114	0.126	0.100
PC2	0.338	0.486	0.300	0.022	−0.112	−0.079	−0.229	−0.242	−0.092	−0.249

Notes: RGP: relative germination potential; RGR: relative germination rate; RGI: relative germination index; RVI: relative vigor index; RSL: relative shoot length; RRL: relative root length; RSFW: relative shoot fresh weight; RRFW: relative root fresh weight; RSDW: relative shoot dry weight; RRDW: relative root dry weight.

A single component score is insufficient to make a comprehensive assessment of all germination traits influenced by priming treatments under salt stress. A comprehensive component score (Y) can be obtained from the contributions of the two components by calculating the weighted average, that is Y = 0.83Y1 + 0.17Y2.

Y1, Y2, and Y for each treatment are shown in Table 5. Based on their Y-values, the efficacies of different seed priming treatments in promoting sorghum germination under salt stress were 50CaCl_2_ > 100CaCl_2_ > 150CaCl_2_ > 150KCl > 10%PEG > HP > 150NaCl > 30%PEG > 50KCl > 100NaCl > 100KCl > 20%PEG > 50NaCl.

**Table 5:** Comprehensive component scores (Y) and order of priming treatment efficacy under saline conditions.

Priming treatments	Y1	Y2	Y	Order of priming efficacy
HP	1.72	-0.04	1.42	6
50NaCl	1.53	-0.82	1.13	13
100NaCl	1.46	-0.07	1.20	10
150NaCl	1.68	-0.21	1.36	7
50KCl	1.65	-0.27	1.32	9
100KCl	1.48	-0.20	1.19	11
150KCl	1.87	-0.26	1.51	4
50CaCl_2_	2.51	-0.36	2.02	1
100CaCl_2_	2.46	-0.77	1.91	2
150CaCl_2_	2.35	-0.35	1.89	3
10%PEG	1.87	-0.68	1.44	5
20%PEG	1.40	0.02	1.17	12
30%PEG	1.62	0.01	1.35	8

### Cluster analysis

**Fig 3:**
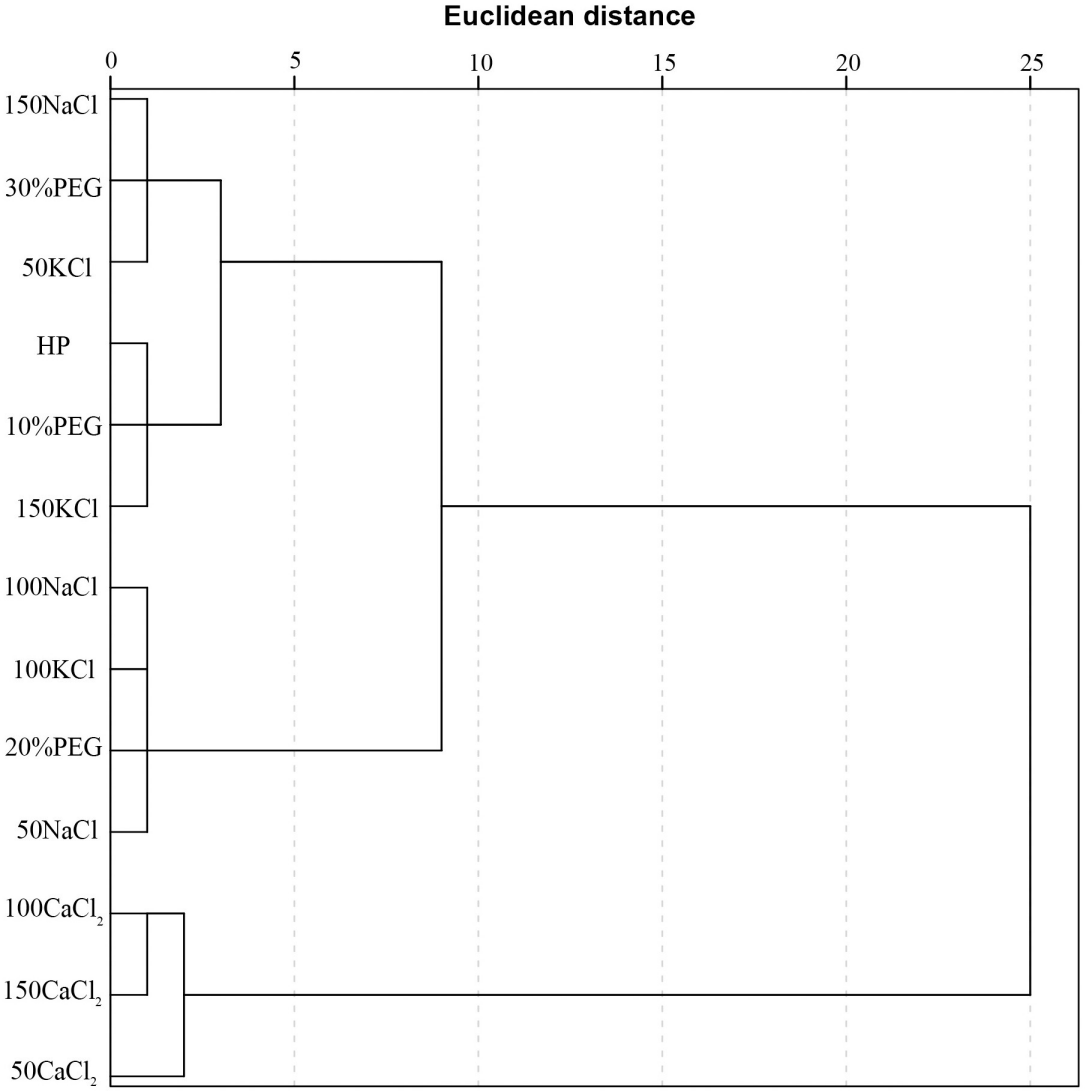
Dendrogram of 13 seed priming treatments based on Y-values using Ward’s method of systematic classification.

Cluster analysis was performed based on the Y-values obtained from the principal component analysis, and the 13 priming treatments were divided into three categories using Ward’s method of systematic classification [38] (Fig 3). The first group comprised the 50CaCl_2_, 100CaCl_2_, and 150CaCl_2_ treatments, which had an optimal effect on seed germination under saline conditions. The second group promoted germination under saline conditions to a lesser extent and comprised the 150KCl, 10%PEG, HP, 150NaCl, 30%PEG and 50KCl treatments. The third group comprised the 100NaCl, 100KCl, 20%PEG, and 50NaCl treatments, which had a minimal effect on seed germination under salt stress.

## Discussion

Sorghum growth is easily inhibited by salt stress, especially during the germination stage [37]. In this study, sorghum germination was significantly inhibited by salt stress: germination potential, germination rate, germination index, and vigor index were all reduced, and seedling growth was inhibited (S1 Table). There are various reasons for delayed and repressed seed germination under salt stress. First, salinity reduces the osmotic potential of the external medium, limiting the seedlings’ capacity for water absorption and thereby inhibiting cell growth [39]. Second, salt stress may reduce the efficiency of seed reserve mobilization during germination by various mechanisms, such as the perturbation of protein structure [23]. In addition, seed respiration may also be enhanced, thereby reducing the levels of reserve substances available for seedling growth [40].

Seed priming is an effective way to alleviate the inhibition of seed germination and seedling growth by salt stress [30]. In this study, although the effects of different priming agents differed, all promoted the germination of sorghum seeds under salt stress to some extent (S2 Table). Iqbal et al. [41] reported that seed priming with NaCl, KCl, and CaCl_2_ alleviated salt stress damage, thereby increasing shoot fresh weight and dry weight in maize. Similarly, Abraha et al. [42] showed that seed priming with 5g/L NaCl significantly improved early seedling growth under salt stress in maize. Likewise, seed priming with PEG-6000 alleviated the adverse effects of salt stress on seed germination and early seedling growth in common vetch [43]. Seed priming with distilled water increased the germination index, root and shoot length, and dry weight and promoted the germination and normal seedling growth of maize under salt stress [44]. These results show that a variety of substances can be used as seed priming agents and can promote seed germination under saline conditions, consistent with the findings of this study.

Priming treatments reduced salt stress damage, and it is interesting that their effects on seed germination potential, germination index, and vigor index were greater than their effects on final germination rate (S2 Table), indicating that priming mainly improved seed vigor rather than the number of germinated seeds under salt stress. Correlation analysis showed that the germination rate was not significantly correlated with seedling growth traits. By contrast, traits (RGP, RGI and RVI) associated with seed vigor were strongly correlated with some seedling growth traits, suggesting that seedling growth was closely related to seed vigor. In other words, seed priming promoted the establishment of strong seedlings under salt stress primarily by improving seed vigor. In addition, priming had a greater positive effect on root traits than on shoot traits. This observation is consistent with the data of Farooq et al. [45], Ghobadi et al. [46], Azooz [47], and Hussain et al. [48] and may be due to a priming-triggered metabolic process that occurs before seedling emergence and prepares the seeds for radicle protrusion [49,50]. In addition, seed priming reduces physical barriers of the endosperm during imbibition, repairs membrane damage, improves the growth of immature embryos, and leaches germination inhibitors to promote radicle growth [1,51,52].

Different priming treatments promoted sorghum germination to different extents under salt stress, and principal component analysis clearly indicated that CaCl_2_ priming was superior to other treatments based on its positive effects on germination traits. The priming treatments were divided into three categories by cluster analysis, and CaCl_2_ priming treatments were again classified as the most effective. Mitigation of abiotic stress by seed priming with CaCl_2_ has also been reported in Farooq et al. [45], Issam et al. [53], and Bismillah et al. [54]. Calcium is one of the main essential nutrients required for plant growth, development, and stress tolerance [55]. Calcium ions play an important role in the regulation of plant metabolism, and free calcium ions inhibit the influx of extracellular sodium ions and maintain the intracellular potassium and sodium ion balance [56], reducing sodium ion toxicity and improving salt tolerance during germination. In addition, calcium signal transduction has a role in plant acclimation to salt stress. When plants are exposed to salt stress, the concentration of intracellular calcium ions increases rapidly [57], and Köster et al. [58] pointed out that calcium ions coordinate the response to salt stress at the cellular and organismal levels. Sodium and potassium ions do not play the same physiological role as second messengers [57]. These effects of calcium ions may explain why calcium chloride priming was the best treatment for sorghum seed germination under salt stress. Jafar et al. [59] reported that although different priming treatments improved the salt tolerance of wheat, priming with CaCl_2_ was the most effective treatment compared with ascorbic acid, salicylic acid, and kinetin. Afzal et al. [60] showed that seed priming with CaCl_2_ was more effective than NaCl in improving wheat salt tolerance at the germination stage. In this study, priming with only distilled water also had a positive effect. However, the benefits of primed seeds are reduced by prolonged storage and storage conditions such as temperature, seed moisture content, and seed quality [61,62]. The effects of other treatments differed depending on their solution concentrations, perhaps because their different osmotic potentials led to different imbibition rates and, eventually, different priming effects.

In summary, our conclusions are as follows: (1) Sorghum germination was significantly inhibited by salt stress, and this effect could be alleviated to varying degrees by seed priming with HP, NaCl, KCl, CaCl_2_, or PEG-6000, and (2) 50 mM CaCl_2_ was the most effective priming treatment for promoting the germination of sorghum seeds under salt stress. Common and inexpensive seed priming agents were used in this study. However, there are many other seed priming agents and methods, and it is helpful to classify different agents and determine which is the best based on their actual effects on seed germination under salt stress. It is worth mentioning that our work was limited to a single sorghum variety germinated and grown *in vitro*. It will therefore be necessary to continue this work with glasshouse or field trials, bearing in mind that different sorghum varieties may respond differently to these treatments. Further research is also necessary to determine the physiological and molecular mechanisms by which CaCl_2_ improves sorghum germination under salt stress.

## Supporting information

S1 Table The raw data. The table matched to Table 1.
(XLSX)

S2 Table The raw data. The table matched to Table 2.
(XLSX)
